# Role of Fibroblast Growth Factors Receptors (FGFRs) in Brain Tumors, Focus on Astrocytoma and Glioblastoma

**DOI:** 10.3390/cancers12123825

**Published:** 2020-12-18

**Authors:** Alessio Ardizzone, Sarah A. Scuderi, Dario Giuffrida, Cristina Colarossi, Caterina Puglisi, Michela Campolo, Salvatore Cuzzocrea, Emanuela Esposito, Irene Paterniti

**Affiliations:** 1 Department of Chemical, Biological, Pharmaceutical and Environmental Sciences, University of Messina, Viale Ferdinando Stagno d’Alcontres, 98166 Messina, Italy; aleardizzone@unime.it (A.A.); sarascud@outlook.it (S.A.S.); campolom@unime.it (M.C.); salvator@unime.it (S.C.); eesposito@unime.it (E.E.); 2 Istituto Oncologico del Mediterraneo, Via Penninazzo 7, 95029 Viagrande (CT), Italy; dario.giuffrida@grupposamed.com (D.G.); cristina.colarossi@grupposamed.com (C.C.); 3 IOM Ricerca Srl, Via Penninazzo 11, 95029 Viagrande (CT), Italy; caterina.puglisi@grupposamed.com

**Keywords:** brain tumors, astrocytoma, glioblastoma, fibroblast growth factors (FGFs), fibroblast growth factors receptors (FGFRs), Fisogatinib, Futibatinib

## Abstract

**Simple Summary:**

Considering the high mortality rate and the increasing spread of brain tumors both in adulthood and in childhood, we explore the role and involvement of fibroblast growth factors receptors (FGFRs) in two specific types of brain tumors: astrocytoma and glioblastoma. Fibroblast growth factors (FGFs) and their receptors (FGFRs) regulate pivotal cellular processes during embryonal development of the CNS, and as a survival mechanism for adult neurons and astrocytes. Moreover, interactions between the neural cell adhesion molecules NCAM and N-cadherin with FGFR are important for a number of developmental events and have also been implicated in tumor progression. Thus, this review provides an overview on the biological mechanisms of FGFRs related to oncogenesis and a new point of view for future preclinical and clinical studies to develop increasingly targeted therapies.

**Abstract:**

Despite pharmacological treatments and surgical practice options, the mortality rate of astrocytomas and glioblastomas remains high, thus representing a medical emergency for which it is necessary to find new therapeutic strategies. Fibroblast growth factors (FGFs) act through their associated receptors (FGFRs), a family of tyrosine kinase receptors consisting of four members (FGFR1–4), regulators of tissue development and repair. In particular, FGFRs play an important role in cell proliferation, survival, and migration, as well as angiogenesis, thus their gene alteration is certainly related to the development of the most common diseases, including cancer. FGFRs are subjected to multiple somatic aberrations such as chromosomal amplification of FGFR1; mutations and multiple dysregulations of FGFR2; and mutations, translocations, and significant amplifications of FGFR3 and FGFR4 that correlate to oncogenesis process. Therefore, the in-depth study of these receptor systems could help to understand the etiology of both astrocytoma and glioblastoma so as to achieve notable advances in more effective target therapies. Furthermore, the discovery of FGFR inhibitors revealed how these biological compounds improve the neoplastic condition by demonstrating efficacy and safety. On this basis, this review focuses on the role and involvement of FGFRs in brain tumors such as astrocytoma and glioblastoma.

## 1. Introduction

**Figure 1:**
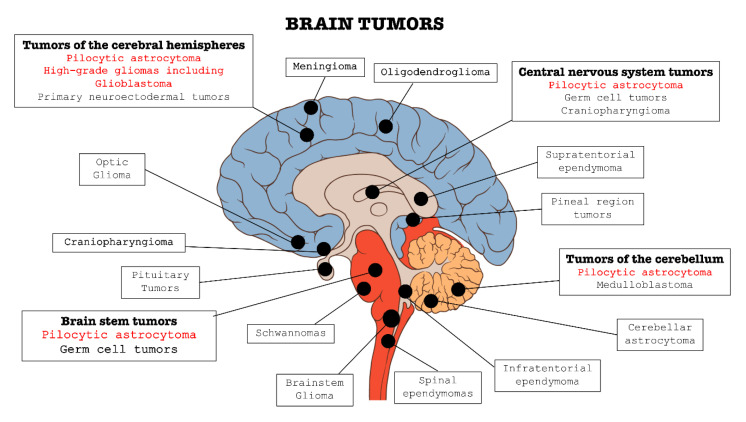
Classification of the main brain tumors; the tumors involved in this review are highlighted in red.

In the context of central nervous system (CNS) disorders, tumors are certainly one of the most widespread and lethal pathologies. These types of cancers affect the CNS in all its parts: brain, bone marrow, and cerebellum (as summarized in Figure 1). Brain tumors are classified into two categories: primary tumors, which originate and develop directly in the central nervous tissue, and secondary tumors, or metastases, which derivate from tumors cells present in other organs, such as lung, breast, gastrointestinal tract, etc. and subsequently spread to nerve tissue [1,2,3].

In the last years, there has been a progressive upsurge in brain tumors incidence; in particular, this increase was most significant in the over 65 age group, and higher in men than women [4]. The manifestations of a brain tumor depend mainly on its location and size [4] that can lead to compression, infiltration of healthy tissue, increasing intracranial pressure, and cerebral edema [5,6].

The onset of symptoms is usually insidious, may not be specific, and include headache, nausea, vomit, mental changes, balance disorders, speech disorders, lack of strength of the limbs, or sensitivity disorders [7,8,9], while an acute vascular complication, e.g., stroke, is a less frequent manifestation [10].

Pharmacological therapy changes considerably according to the age of the patient and the site of the neoplasm [11,12].

Chemotherapy may be useful in some types of brain tumors even if this therapeutic approach is hampered by the presence of the blood–brain barrier (BBB) [13,14]. BBB acts as a kind of natural filter that limits the passage of substances, and therefore also drugs, from the blood to the brain tissues [15]. Among the drugs capable of crossing BBB, the most used are alkylating agents such as Temozolomide and drugs belonging to the Nitrosoureas family such as Lomustine and Carmustine [16,17]. Often, more effective results have been obtained by combining drug therapy with radiotherapy [18]. In fact, the combination of the three drugs Lomustine, Procarbazine, and Vincristine [19] with radiotherapy contributed to significantly prolong the survival of patients with low-grade gliomas [20,21]. Over the years, radiotherapy (RT) has increasingly assumed a fundamental role in the treatment of primary brain tumors as well as metastases [22]. Indeed, thanks to advances in imaging and radiotherapy techniques, it has been possible to make a more precise localization of the tumor, thus allowing a reduction in the volume of irradiated healthy brain tissue [22]. All this led to a reduction in long-term toxicity due to radiotherapy, but with the same results in terms of efficacy.

In addition to these, surgical treatment represents one of the most valid options in the case of both primary tumors and metastases [23,24]. Moreover, the process of carcinogenesis in the brain involve various pathways and molecular mechanisms, many of which are still under study and deepening. Recent articles, for example, highlight the role of receptors such as interleukin 13 receptor alpha 2 (IL-13RA2) as a tumor-associated receptor over-expressed in most patients with glioblastoma as well as the overexpression of EphA2 and EphA3 receptors [25].

Therefore, the in-depth study of further receptor systems could help to understand the etiology of gliomas so as to obtain notable advancements in more effective target therapies. In the last decade, several studies have investigated the role of fibroblast growth factors receptors (FGFRs) in the background of brain tumors, thus such evidence could provide a further useful point of view in the treatment of tumors including astrocytoma and glioblastoma.

## 2. Fibroblast Growth Factors (FGFs) and Fibroblast Growth Factors Receptors (FGFRs)

Fibroblast growth factors (FGFs) are key regulators of tissue development and repair acting through their associated receptors (FGFRs) [26].

In 1973, the first FGF was discovered in pituitary extracts, and it was found to be widely expressed in cells and tissues [27,28]. Primarily, acidic FGF (FGF1) and basic FGF (FGF2) were isolated from the brain and pituitary gland as growth factors for fibroblasts [27,29]. Since then, they have been identified in both vertebrates and invertebrates, and currently 22 FGFs are known in humans [30].

FGFs are involved in various biological functions, as demonstrated by both in vitro and in vivo studies [31,32], and have their specific role in mitogenesis, cell migration, differentiation, angiogenesis, and wound healing [33]. The activity of FGFs, and their physiological role, is expressed through the bond with FGFRs, thanks to which they regulate the processes of cell growth, thus controlling events such as the modeling of the mesoderm in the initial embryo across the development of multiple organ systems [27].

Given that the biological activity of FGFs is expressed through their link with FGFRs, it is useful to identify the involvement of FGFRs in biological systems, cross-cascade intercommunication among signal transduction pathways, and their implication in human disease.

FGFRs are a family of tyrosine kinase receptors consisting of four members, FGFR1, FGFR2, FGFR3 and FGFR4, with 22 known ligands [34,35] and encoded by four different genes: Flg, Bek, Cek-2, and Frek. These factors share a core homology domain that consists of about 120 amino acid residues that assume a globular β-trefoil structure that consists of 12 β-strands arranged into three similar sets of four-stranded β-sheets. This core domain is flanked by divergent amino-terminal and carboxyterminal sequences that account in part for the selectivity and specificity of the growth factors [36].

These receptors are transmembrane proteins with their own well-defined structural characteristics [37]. Specifically, the extracellular portion consists of a hydrophobic signal sequence, e.g., IgG domains [38], and an acidic region (acid box) essential for binding to heparin [39], comprising four and eight acidic amino acids varying in the various FGFR receptors [40]. In the C-terminal portion of the receptor, on the other hand, there are transmembrane and intracellular regions; precisely, the latter includes a juxta-membrane region, two kinase domains, and a region between kinase domains (called kinase insert), as well as a carboxy-terminal region that contains the autophosphorylation sites, thus allowing interaction with specific substrates [41].

Furthermore, the presence of divalent cations (including calcium, manganese, etc.) in the acidic alloy region contributes to the achievement of an optimal conformation of optimal FGFR [42], necessary for the high-affinity receptor/heparin-like glycosaminoglycans (HLGAG) interaction and consequently for the binding of the receptor to the ligand [43]. As with all tyrosine kinase receptors, FGFRs also have the ability to transmit various extracellular signals within the cell by activating some signal transduction pathways [44,45]; the complex signal transduction mediated by FGFRs reflects the various physiological functions that these factors regulate [45].

Activation of FGFRs, at the plasma membrane, involves the transduction of biochemical signals through a process known as dimerization. Receptor dimerization is essential for activation, as it brings the two tyrosine kinase domains into close proximity, thus allowing each other to phosphorylate the tyrosine in their activation circuits [46,47]. This process activates kinases, which in turn bind adapter proteins and phosphorylated cytoplasmic substrates, thus triggering downstream signaling cascades that control cell growth and differentiation [48].

Once activated, the receptor can in turn phosphorylate and activate various signal transducer molecules, both directly and indirectly such as AKT and the anti-apoptotic pathway dependent on this, as well as the Ras-Raf-MEK-ERK pathway [49,50].

**Figure 2:**
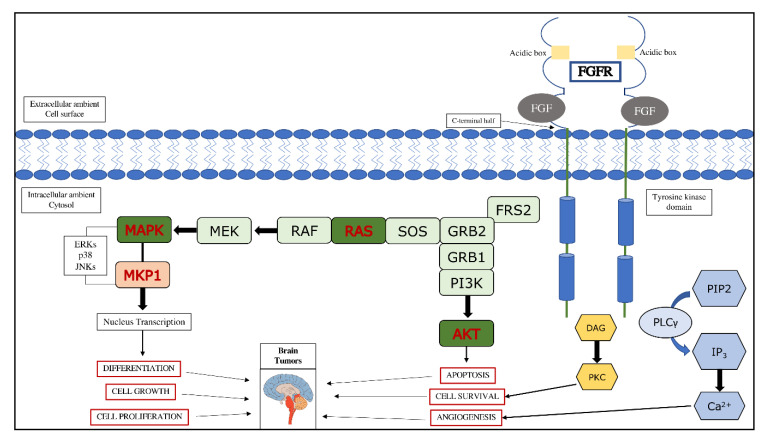
FGF/FGFR signaling pathway and its involvement in brain tumors. FRS2, fibroblast growth factor receptor substrate 2; GRB, growth factor receptor bound protein 2; SOS, son of sevenless; RAS, monomeric G-protein; RAF, kinase; MEK, kinase; MKP1, MAP kinase phosphatase; PIP2, phosphatidylinositol (4,5)-bisphosphate; PLCγ, phospholipase C γ; IP3, inositol triphosphate; DAG, diacylglycerol; PKC, protein kinase C.

Thus, based on their mechanism of action, it is understandable how these receptors are involved in the processes of oncogenesis, as summarized in Figure 2.

### 2.1. FGFRs and Cell Adhesion Molecules (CAMs)

FGFR1–4 can be activated by their ligands, FGFs, and by cell adhesion molecules (CAMs) such as the neural cell adhesion molecule (NCAM), L1-CAM, and N-cadherin to induce specific cell responses and fate during development and cancer [51].

In the absence of FGF, FGFR exists in the cell membrane as a monomeric protein that dimerizes upon binding to FGF. FGFR dimerization brings the tyrosine kinase domains of two receptor molecules into close proximity to each other, followed by autophosphorylation of the kinase domains, and thus receptor activation. In contrast to transiently expressed FGFs, CAMs are expressed constitutively, and are thought to activate FGFR only when they are involved in cell–cell adhesion [52].

There is now substantial evidence that some CAM functions require the activation of specific second messenger signaling cascades in cells, for example the ability of NCAM, N-cadherin, and L1 to stimulate axonal growth is dependent on the tyrosine kinase activity of the fibroblast growth factor receptor (FGFR) in neurons [53,54,55,56,57,58]. FGFRs contain about 20 amino acid motifs within the D2 domain that shares sequence homology with functional motifs present in NCAM and N-cadherin [59]. This “CAM homology domain” (CHD) forms a contiguous sequence with the acid box and antibodies raised against the acid box or the CAM homology domain inhibit CAM stimulated neurite outgrowth [54]. Importantly, synthetic peptide mimetics of motifs from this region of the FGFR also inhibit the CAM responses [54]. This formed the basis for the suggestion that the CAMs might directly interact in cis with this region of the FGFR [59].

Moreover, there is now increasing evidence that CAM interactions with the FGFR are not only important for neuronal function but also for contact dependent survival of some cell types [60,61] and for the development and progression of some cancers [62,63,64].

Recent evidence demonstrates that neuronal cadherin (*N-cadherin*) is expressed in various cell types, but its highest level is detected in neuronal and mesenchymal cells, where it coordinates cell migration and proliferation [65]. The functional interaction between N-cadherin and FGFRs was demonstrated in numerous cells, where N-cadherin was shown to activate FGFRs and receptor-downstream signaling [54,55,57].

Formation of N-cadherin complexes with FGFR1 in cancer cells causes decreased internalization and lysosomal degradation of FGFR1 and sustained receptor signaling via MAPKs. Thus, N-cadherin may promote invasiveness of cancer cells not only by regulating cell–cell interactions but also by affecting FGFR1 levels and activity [62,66,67]. N-cadherin stabilizes FGFR1 and decreases its internalization, thus promoting invasion in breast cancer cells, and N-Cadherin/FGFR crosstalk promotes neurite outgrowth [65]. Silencing of N-cadherin results in the accelerated FGFR1 degradation, whereas overproduction of N-cadherin is accompanied by increased levels of FGFR1. It is now speculated that N-Cadherin/FGFR1 interactions could constitute a positive feedback loop in glioblastoma cancer stem cells (GSCs) through subsequent induction of N-cadherin and FGFR1 expression.

In addition, neural cell adhesion molecules (*N-CAMs*) are cell surface glycoproteins involved in axonal growth, cell differentiation and are implicated in cancer development [68,69]. N-CAMs contain five Ig-like domains and two FN3 domains in their extracellular region. The functional interplay between FGFRs and N-CAMs in neurite outgrowth was initially demonstrated by Williams et al. [54]. Subsequent studies confirmed a direct interaction of N-CAMs and FGFRs in different types of cells, including cancer cells [63,70,71,72,73]. N-CAMs bind to FGFR1–3, but not to FGFR4, and these interactions depend on the receptor splice variants [72]. Binding of N-CAMs to FGFRs results in activation of the receptor and initiation of signaling cascades. The N-CAMs-FGFRs interplay is important for neuronal tissue development but is also implicated in cancer like glioblastoma.

Furthermore, polysialic acid-NCAM (PSA-NCAM) has been described as a marker of glioblastoma patient prognosis [74]. Targeting expression of PSA-NCAM in C6 glioma cells resulted in increased levels of Olig2, a transcription factor associated with GSCs [75]. Furthermore, the L1-CAM/FGFR1/Anosmin-1 complex regulates neurite branching [76,77,78] and L1-CAM-mediated FGFR1 transactivation induces glioma cell proliferation and motility [79].

In summary, FGFR activity can be modulated non-canonically by other cell surface proteins, resulting in the activation of intracellular signaling pathways and cell responses associated with FGFR signaling.

### 2.2. Involvement of FGFRs Subtypes in Cancer

Among the various mechanisms that connect FGFRs and the related oncogenesis, the following are certainly included: activating or “driver” mutations [80,81,82] with consequent cell growth and survival, neoangiogenesis, and acquired resistance to other anticancer therapies [45,83]. Carcinogenesis may also be due to the multiple somatic aberrations to which FGFR is subjected. Especially, the overexpression of the receptor can be the consequence of a gene amplification or variations in post-transcription processing; point mutations can be the cause of the activation of the constitutive receptor or a decrease in the sensitivity to binding of the ligand; translocations can produce fusion proteins with constitutive activity; and isoform switching and alternative splicing can reduce the specificity of FGFs [83,84]. These main oncogenic aberrations represent characteristics that make FGFR an ideal therapeutic target for the treatment of many forms of malignant tumors.

Chromosomal amplification processes involving FGFR1 were detected in 10% of breast cancers, especially in estrogen receptor positive cancers [83]. Recently, FGFR1 amplifications have also been found in non-small cell squamous lung tumors (SqCLC) [85] as well as oral squamous cell carcinoma [86], ovarian cancer [87], bladder cancer [88], and rhabdomyosarcoma [89]. In addition, a chromosomal abnormality involving the FGFR1 gene on chromosome 8p11 in myeloproliferative syndrome (EMS) was also detected [90].

Mutations of FGFR2 are present in 12% of endometrial carcinomas [91], and approximately 10% of gastric cancer cases are associated with amplification and/or mutation of FGFR2 [92]; moreover, the degree of amplification is closely related to prognosis.

Multiple FGFR2 dysregulations have also been detected in breast cancer [93], lung cancer [94,95] (both adenocarcinoma and squamous cell carcinoma), and intrahepatic cholangiocarcinoma, in which fusions of FGFR2 constitute an oncogenic potential for this aberration [96].

Studies and analyses carried out on FGF3 have also highlighted its role in the pathogenesis of cancer. In particular, this receptor subtype is involved in bladder cancer [97] and salivary adenoid cystic cancer [97], in which significant amplifications of FGFR3 have been found. Mutations or translocations of FGFR3 are instead implicated in cervical cancer [97], multiple myeloma [98], and bladder cancer [45]. For FGFR4, amplifications and/or mutations of this receptor subtype have been identified in 7–8% of patients with rhabdomyosarcoma [45,99]. Furthermore, some preclinical studies have shown the overexpression of FGFR4 in cancers of the prostate [100], colon [101], and liver [102], in which this receptor could have a potential role in the development and progression of tumors [83].

## 3. Role of FGFRs in Brain Tumors

Primary CNS tumors include a diverse set of pathological entities; however, despite the above-mentioned generic classification, it is more appropriate to distinguish brain tumors on the basis of their origin; a first distinction can be made by considering “non-glial tumors” and “glial tumors”.

These last types of tumors originate from glial cells and represent the most common form of all CNS tumors, representing 81% of malignant brain tumors [103]. They are classified into various subtypes, distinguished according to the cell type from which they originate and the degree of differentiation and/or malignancy [104,105]. Among the most common gliomas, we have astrocytomas (originating from astrocytic cells) including glioblastoma, oligodendrogliomas (from oligodendroglial cells), and ependymomas (from ependymal cells) [106].

Given that FGFRs play a significant role in cell proliferation, survival, and migration, as well as angiogenesis, their gene alteration is certainly correlated with the development of most common pathologies, including several types of cancer such as breast, bladder (specifically, urothelial cell carcinoma), lung (small cell length carcinoma and non-small cell lung carcinoma), prostate, and multiple myeloma [107]. In this review, we focus on their involvement in brain tumors, with particular attention on astrocytoma and glioblastoma, as summarized in Table 1.

### 3.1. Role of FGFRs in Astrocytoma

Astrocytoma is a tumor that originates from a specific type of glial cell: “astrocytes” [112]. Astrocytes are the most differentiated glial cells, characterized by elaborate radially symmetrical branches, which attribute to them the characteristic star shape [113]. On the basis of their geometry, they are distinguished into protoplasmic forms, when short and rare extensions are present; fibrous forms, when they have numerous long and thin cytoplasmic; or radial extensions, of elongated shape and distributed perpendicular to the axis of the ventricles [114]. Astrocytomas represent the most common forms of gliomas [115], representing 80% of malignant tumors of CNS [116]; specifically, low-grade gliomas are more frequent in young ages [117,118], while anaplastic or malignant gliomas generally have a later onset [119], even if there are exceptions.

There are several classifications proposed, in general low or high malignancy astrocytomas are distinguished while in particular a distinction is made based on four degrees: I, II, III, and IV [120].

The main categories of astrocytomas are: pilocytic astrocytoma and subependymal giant cell (grade I), diffuse astrocytomas (grade II), pleomorphic xanthastrocytomas (grades II and III), anaplastic astrocytoma (grade III), and glioblastoma (grade IV) [121]. These various degrees of histological variability correspond to various degrees of malignancy, which is given both by the rapidity of growth and by the ability to reform themselves after surgical removal [104].

Therefore, the assessment of gradation is an important parameter for both prognosis and therapy [104].

The latest revision proposed by the World Health Organization (WHO) classifies gliomas by integrating the data of conventional histological analysis with molecular information obtained through specific genetic analyses [122]. In particular, it is highlighted how the presence or absence of a mutation in the isocitrate dehydrogenase (IDH) 1/2 gene [123,124,125], deletion of chromosome arms 1p and 19q (1p/19q codeletion), and mutations in the TERT promoter are determining factors in establishing a specific histomolecular subtype [126,127].

The causes that lead to the formation and development of astrocytoma are still little known and in continuous analysis. However, it is recognized that defects related to chromosomal and genetic mutations play a decisive role in the uncontrolled growth of brain cells, involving multiple mechanisms and pathways, in which FGFRs also contribute [108].

This statement is confirmed in a study by Lew et al. [128], in which the oncogenic role played by FGFRs receptors, and in particular by FGFR1, is well highlighted. Specifically, the precise sequence of FGF receptor autophosphorylation is kinetically driven and is disrupted by oncogenic mutations. Such involvement of FGFRs in oncogenesis processes made these receptors one of the most promising targets for FGFR-derived cancer therapies. Thus, drugs targeting FGFRs could be an effective therapeutic approach for cancers [129].

Moreover, a study by Sie et al. [130] proved how pediatric low-grade astrocytoma cell line showed high percentages of cells expressing FGFR1 (34–51%) compared to isotype controls. The study showed how the inhibition of FGFR1 decreased tumor cell viability, thus highlighting the importance of environmental growth factors in developing tumor escape towards RTK inhibitors [130]. Similar results were reported by Trisolini et al. [131], which highlight the frequent FGFR1 mutation in optic-pathway pilocytic astrocytomas, revealing FGFR1 as an excellent candidate for anti-FGFR therapies in patients [131].

The involvement of FGFR1 and FGFR3 in pilocytic astrocytoma was also confirmed by Lehtinen et al. [132]. In this study, immunohistochemical analysis revealed how moderate-to-strong FGFR3 expression was detected predominantly in non-pediatric patients [132]. In addition to this, strong expression of the FGFR3 protein is indicative of FGFR3 fusions and may serve as a clinically applicable predictive marker for FGFR inhibitor-based treatment regimens [133].

Further evaluations on FGFR3 and in particular on gene fusions of FGFR3-TACC3 (F3-T3) were carried out by Frattini et al. [134], elucidating the oncogenic circuitry activated by F3-T3, showing that F3-T3 positive tumors rely on mitochondrial respiration, and highlighting this pathway as a therapeutic opportunity for the treatment of tumors with F3-T3 fusions [134].

FGFR-TACC fusions generate potent oncogenes that combine growth-promoting effects with aneuploidy through the activation of still unclear intracellular signaling mechanisms [135]. In relation to this, clinical data show promising effects in cancer patients hosting FGFR-TACC fusions and treated with FGFR inhibitors [135], therefore future insights could lead to encouraging results.

### 3.2. Role of FGFRs in Glioblastoma

Gliomas are malignant primary brain tumors, among which glioblastoma has the worst prognosis [136]. According to the most recent WHO guidelines, it is classified as a grade IV diffuse astrocytoma [136]. Glioblastoma is a particularly aggressive type of cancer affecting the glial cells, in particular astrocytes, which have a supporting role in CNS [137]. Furthermore, it represents about 45.2% of all malignant CNS tumors [138,139] with an incidence of 5–6 cases per 100,000 people [140].

Glioblastoma is characterized by uncontrolled proliferation, angiogenesis, invasiveness, and necrosis [141], and it can develop de novo or through the malignant progression of lower-grade astrocytomas [142]. Numerous risk factors leading to the development of glioblastoma have been identified as genetic factors and environmental factors including exposure to therapeutic ionizing radiation, pesticides, and smoking [140]. Glioblastoma is usually described in two different clinical forms, primary and secondary; primary glioblastoma is the most common form (about 95%) and arises typically de novo, within 3–6 months, in older patients, while secondary glioblastoma arises from prior low-grade astrocytomas (over 10–15 years) in younger patients [142]. The majority of glioblastoma tumors occur in the frontal lobes of the supratentorial compartments, in particular the temporal and parietal lobes, but they also occur in the cerebellum and spinal cord. In the last decade, many studies have been conducted to understand the role of genetic mutations and microenvironment in glioblastoma tumorigenesis [109,110,111,143].

Glioblastoma exhibits several cytogenetic abnormalities involving the loss or structural rearrangement of loci on chromosomes 9, 10, and 17 [144,145,146]. Many studies have revealed that somatic mutations of FGFRs signaling are among the most frequent molecular alterations that occur in glioblastoma, being involved in progression and growth tumor [147,148]. Deregulation of FGFR signaling is frequently observed in many types of cancer including glioblastoma, promoting the development and growth of cancers cell [149]. Gene expression analysis revealed profound heterogeneity of FGFR1–4 expression in glioblastoma patients [147]. Altered FGFR expression in astrocytes can lead to glioblastoma progression due to the activation of mitogenic, migratory, and antiapoptotic responses [147]. Several studies have reported that FGFR1 and FGFR2 gene amplification, abnormal activation, or single nucleotide polymorphisms (SNPs) have a key role in glioblastoma progression [135,147]. In this context, it has been shown that FGFR1 expression increases as the tumor progresses from benign to malignant, whereas FGFR2 levels in human gliomas gradually diminish [150]. Moreover, a recent report found that FGFR3 and FGFR4 are also expressed in invasive glioblastoma cells [98]. Scientific evidence reveals that human glioblastoma is also characterized by oncogenic fusions involving the members of the FGFR3 and FGFR1 tyrosine kinases (TKs) to the transforming acidic coiled-coil (TACC) proteins, in particular TACC3 and TACC1 [135], necessary to promote cell division [149,151]. The fusion between FGFR3 and TACC3 genes generates an oncogenic FGFR3 form [149]. It was reported that the ectopic expression of FGFR3-TACC3 fusion affects about 3% of glioblastoma patients, and its activation through dimerization and transphosphorylation of kinase domain contribute to carcinogenic events closely related to glioblastoma progression [135,152].

Furthermore, it is important to highlight that the mechanism of autocrine stimulation, in the context of glioblastoma, contributes to cell growth and invasion.

The high invasiveness of tumor cells remains one of the most critical challenges in the clinical management of patients with glioma [153] and in particular for glioblastoma patients.

This invasion of glioma cells is stimulated by both autocrine and paracrine factors which act on a wide range of cell surface-bound receptors. Among the key signaling elements that mediate receptor-initiated signaling in regulating glioblastoma invasion, there are Rho family GTPases [154], but FGFs play also their role, as in the case of basic fibroblast growth factor (FGF2, also called bFGF) [155,156,157].

This process of self-renew and tumor proliferation of glioblastoma cells by FGFs were also described by Allerstorfer et al. [158]. They demonstrated the contribution of FGF5 in the malignant progression of human astrocytic brain tumors by both autocrine and paracrine mechanisms. Moreover, their data indicate FGF5 exerts oncogenic activities in astrocytic brain tumors by promoting growth, survival, and migration effects on tumor cells and by supporting neoangiogenic processes [158]. siRNA-mediated FGF5 downregulation thus leads to a significant reduction in glioblastoma cell proliferation [158]; therefore, the silencing of this factor represents a promising target for therapeutic interventions in human glioblastoma.

Accordingly, a greater understanding of the molecular mechanisms that control invasion of glioblastoma cells may lead to the identification of future molecular targets for therapeutic intervention in this devastating disease.

## 4. FGFRs Inhibitor

The inhibition of FGFR could be a viable therapeutic option for this type of brain tumor [150]. Given the multiple functions performed by FGFRs, the development of molecules that interact with these receptors is one of the central focuses of research. In particular, the recent discovery of two FGFR inhibitors, fisogatinib and futibatinib, revealed how these biological compounds improve the neoplastic condition with demonstrated efficacy and safety.

### 4.1. Fisogatinib

**Figure 3:**
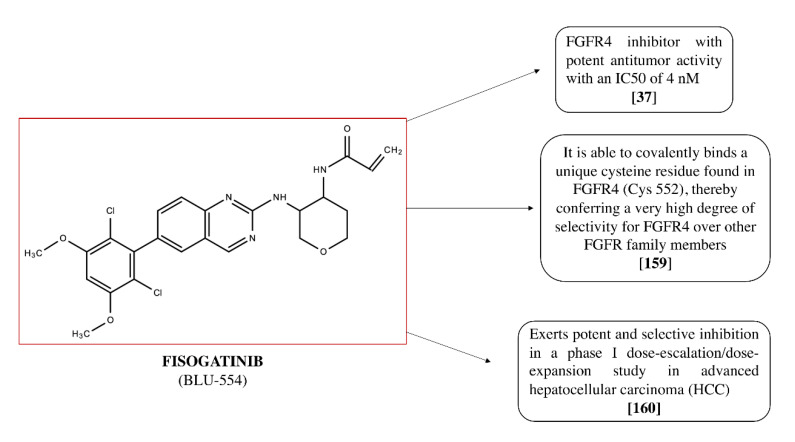
Chemical structure of Fisogatinib (BLU-554) and its properties.

Fisogatinib (BLU-554; see Figure 3), a quinazoline derivative, is an FGFR4 inhibitor with a high bioavailability after oral administration, through which it exerts a potent antineoplastic activity. Indeed, after oral administration, fisogatinib specifically inhibits FGFR4 and the additional binding of the ligand FGF19 to FGFR4.

The potent activity of fisogatinib (IC_50_ of 4 nM) [37], together with its high selectivity, results in a significant anti-tumor activity, as demonstrated by clinical studies [159,160]. Fisogatinib is able to covalently bind a unique cysteine residue found in FGFR4 (Cys 552), thereby conferring a very high degree of selectivity for FGFR4 over other FGFR family members [159].

Recent data highlight the potent and selective inhibition of fisogatinib in a phase I dose-escalation/dose-expansion study in advanced hepatocellular carcinoma (HCC). This study demonstrated that FGFR4 is a promising therapeutic target and how fisogatinib was well tolerated and clinically active in advanced HCC. In particular, it validated the oncogenic driver role of the FGFR4 pathway in HCC and the use of FGF19 as a biomarker for patient selection, demonstrating favorable pharmacokinetics properties [159,160]. In addition, further analyses were carried out to investigate the pharmacokinetics of fisogatinib. The study performed on mice carried out by Sparidans et al. [161] discovered that the accumulation of fisogatinib in the brain is substantially limited by ABCB1 P-glycoprotein in the BBB, while oral availability of fisogatinib is considerably restricted by CYP3A activity [161]. The whole of these few but precious data constitute a considerable aid for optimizing the clinical efficacy of this promising compound.

### 4.2. Futibatinib

**Figure 4:**
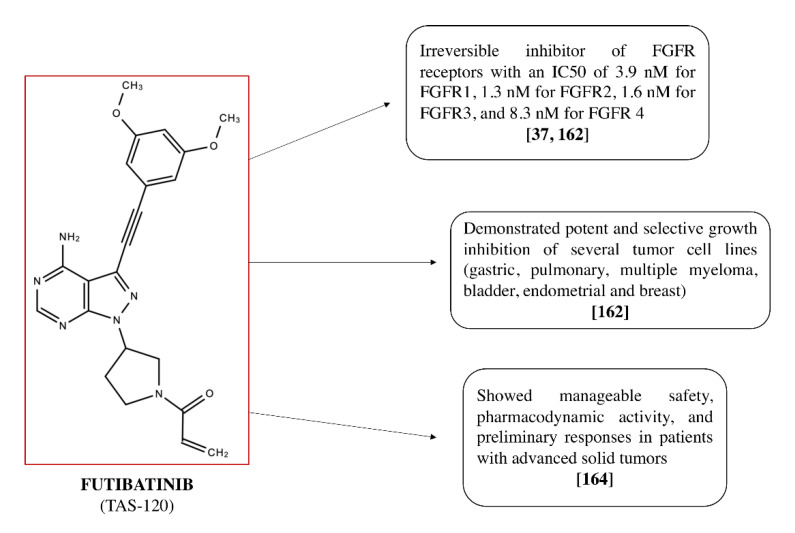
Chemical structure of futibatinib (TAS-120) and its properties.

Futibatinib (TAS-120; see Figure 4) is a pyrazolo[3,4-d]pyrimidine derivative, an irreversible inhibitor of FGFR receptors, highly selective and with good oral bioavailability. It has an IC_50_ of 3.9 nM for FGFR1, 1.3 nM for FGFR2, 1.6 nM for FGFR3, and 8.3 nM for FGFR 4 [37,162].

The selective and irreversible binding of FGFRs, with their consequent inhibition, results in the inhibition of the FGFR-mediated signal transduction pathway and therefore of tumor cell proliferation, thus enhancing potential antineoplastic activity. Futibatinib is currently under phase I/II clinical trials in patients with confirmed advanced metastatic solid tumors harboring FGFR aberrations [163].

Additional information is provided by a study of Sootome et al. [162], in which futibatinib, thanks to its ability to covalently bind the FGFR kinase domain, showed a broad antiproliferative activity in cancer lines and animal tumor models by deregulating FGFR levels. More specifically, futibatinib showed potent and selective growth inhibition of the following tumor cell lines: gastric, pulmonary, multiple myeloma, bladder, endometrial, and breast. Additionally, oral administration of futibatinib resulted in significant tumor shrinkage in tumor xenograft models in both mice and rats, and tumor shrinkage was associated with FGFR inhibition in a dose-dependent manner [162].

Furthermore, futibatinib also demonstrated potent and selective growth inhibition of several tumor cell lines (gastric, pulmonary, multiple myeloma, bladder, endometrial, and breast) having different FGFR genomic aberrations. In addition, its oral administration significantly reduced, in a dose-dependent manner, the volume of the tumor in various xenograft models [162]. The results of this study highlight the broad spectrum of anti-tumor activity of futibatinib, providing a valid rationale for testing futibatinib in clinical trials [162].

The first clinical study performed with futibatinib [164] had as its main objective the evaluation of the safety and pharmacokinetics/pharmacodynamics in advanced solid tumors. The results obtained show that treatment with futibatinib resulted in manageable safety, pharmacodynamic activity, and preliminary responses in patients with advanced solid tumors [164].

### 4.3. AZD4547

AZD4547, *N*-[5-[2-(3,5-Dimethoxyphenyl)ethyl]-2*H*-pyrazol-3-yl]-4-(3,5-diemthylpiperazin-1-yl)benzamide, is a selective FGFR1–3 inhibitor [165]. AZD4547 is an orally bioavailable and highly selective compound. Thanks to its chemical structure and selectivity profile, this compound has been shown to be capable of significantly inhibit FGFR phosphorylation and repressing the proliferation of tumor cell lines via inhibition of FGFR signaling [165]. AZD4547 has high potency against FGFR showing an IC_50_ of 0.2 nmol/L for FGFR1, 2.5 nmol/L for FGFR2, and 1.8 nmol/L for FGFR3; however, AZD4547 has a much lower potency against FGFR4, whose kinase domain is structurally different [165]. AZD4547 demonstrated potent inhibition of proliferation in tumor xenograft models with deregulated FGFR expression as glioblastoma [165]. Relevant information is provided by a study conducted by Singh et al. [149], which demonstrated that oral administration of AZD4547 resulted in prolonged survival of a FGFR3-TACC3-transformed glioma xenograft model compared with mice treated with the vehicle control [149].

Currently, the compound AZD4547 is being studied in several Phase I and II clinical trials, as shown in a study conducted by Andre et al. [166] in patients with recurrent IDH wild-type gliomas with *FGFR1–TACC1* or *FGFR3–TACC3* fusions. Thus, AZD4547 has been shown to promote favorable therapeutic outcomes against a variety of FGFR-deregulated cancer models.

### 4.4. Infigratinib

Infigratinib (NVP-BGJ398 or BGJ398), an N -aryl- N′-pyrimidin-4-yl urea derivative, is a potent and selective FGFR inhibitor [167]. BGJ398 is an orally bioavailable, selective, ATP-competitive FGFR inhibitor with activity against tumor models harboring FGFR alterations, including mutations and aberrations [167]. Infigratinib selectively binds to and inhibits the activities of FGFRs, which may result in the inhibition of tumor angiogenesis and tumor cell proliferation and the induction of tumor cell death. BGJ398 has IC_50_ of 0.9, 1.4, 1, and 60 nM for FGFR1, FGFR2, FGFR3, and FGFR4, respectively, with a predominant activity on FGFR1–3 [168]. A relevant study conducted by Konecny et al. [169] demonstrated that BGJ398 is able to inhibit in vitro cell growth of *FGFR2*-mutant endometrial cancer cell, as well as in vivo in tumor xenograft models. In addition, BGJ398 demonstrated a tolerable safety profile in patients with advanced solid tumors bearing *FGFR* mutations or fusions. Infigratinib is currently under phase I/II clinical trials in patients with solid tumors or hematological malignancies associated to FGFR alterations [168]. Furthermore, Guagnano et al. [168] revealed that BGJ398 significantly inhibits proliferation of different cancer cell lines bearing FGF/FGFR genetic alterations in various solid tumors, namely breast, gastric cancer, and multiple myeloma, showing also antiangiogenic effect. Therefore, on the basis of this scientific evidence, BGJ398 could be considered a valid and alternative strategy for management of solid tumors associated to FGF/FGFR genetic alterations.

## 5. Conclusions

Despite countless scientific advances, current astrocytoma and glioblastoma treatments have not improved the survival rates of patients. Targeted therapies have been shown to have limited efficacy as the pathophysiological mechanisms of brain tumors are still not fully understood. Therefore, the identification of chromosomal mutations and molecular pathways involved in glioma development is an important purpose for oncology research. In this context, FGFR inhibition has found great interest from scientific research in glioma progression. In this context, FGFR inhibitors as fisogatinib and futibatinib, as evidenced in many studies, could represent a potential therapeutic treatment to counteract astrocytoma and glioblastoma growth, which have extremely high mortality due to resistance to currently used therapies.

## 6. Future Perspectives

As for most anticancer drugs, the biodistribution of FGFR inhibitor drugs in intracranial tumors is still unknown due to the lack of pharmacokinetic studies in patients with malignant glioma [170].

This remains a notable shortcoming that prevents clinical success, and therefore further preclinical and clinical studies are needed to learn whether the different FGFR inhibitors are able to achieve adequate therapeutic concentrations in CNS. However, the role of FGFRs in glioma progression suggests the great importance to identify new biological compounds to counteract tumor growth. Consequently, considering the properties of FGFR inhibitors could represent alternative treatments for glioma, alone or in association with chemotherapy drugs generally used to improve the quality of life of patients.

## Figures and Tables

**Table 1:** The subtypes of FGFRs (FGFR1–4) with related molecular weight, residues AA, malignancy type, and inhibitor FGFRs are shown. In addition, i a framework of the common FGFR alterations in brain tumors is also provided. The main mutations of FGFRs, for pilocytic astrocytoma and glioblastoma, are illustrated in the last two columns.

FGFRs	Molecular Weight	Residues AA	Malignancy Type	Inhibitor FGFRs	Common FGFR Alterations in Brain Tumors	Mutation of FGFRs in Pilocytic Astrocytoma	Mutation of FGFRs in Glioblastoma
FGFR1	91.9 kDa [37]	822 [37]	Glioblastomas Low grade brain gliomas [37]	Futibatinib, Infigratinib AZD4547 [37]	FGFR1-TKD [80] FGFR1-TACC1 Fusion [80] FGFR1 hotspot mutations: p.N546, p.K656 [80]	Residues in αA1: N546K (KD1) [108] Residues in αB1: N544K (KD1) [108] Residues in αA1: K655I (KD2) [108] Residues in αB1: K653I (KD2) [108] Residues in αA1: K656D/E/M/N (KD2) [108] Residues in αB1: K654D/E/M/N (KD2) [108] Residues in αA1: T658P (KD2) [108] Residues in αB1: T656P (KD2) [108]	Residues in αA1: N546K (KD1) [81] Residues in αB1: N544K (KD1) [81] Residues in αA1: R576W (KD1) [81] Residues in αB1: R574W (KD1) [81] Residues in αA1: K656E (KD2) [109] Residues in αB1: K654E (KD2) [109]
FGFR2	92.0 kDa [37]	821/822 [37]	Glioblastomas, Low grade brain gliomas [37]	Futibatinib, Infigratinib, AZD4547 [37]	FGFR2-CTNNA3 fusion [80]	Residues in IIIb: K660E (KD2) [108] Residues in IIIc: K659E (KD2) [108]	Residues in IIIb: Q212K (IgII) [109] Residues in IIIc: Q212K (IgII) [109] Residues in IIIb: G463E (JM) [82] Residues in IIIc: G462E (JM) [82]
FGFR3	87.7-88.2 kDa [37]	806/808 [37]	Glioblastomas, Low grade brain gliomas [37]	Futibatinib, Infigratinib, AZD4547 [37]	FGFR3-TACC3 fusions [80]		Residues in IIIb: E468K (JM) [110] Residues in IIIc: E466K (JM) [110] Residues in IIIb: R605Q (KD2) [111] Residues in IIIc: R603Q (KD2) [111]
FGFR4	88.0 kDa [37]	802 [37]	Glioblastomas, Low grade brain gliomas [37]	Fisogatinib [37]			Residues in P22455-1: Q144E (IgI – IgII) [109] Residues in P22455-2: Q144E (IgI – IgII) [109] Residues in P22455-1: R434Q (JM) [109] Residues in P22455-2: R394Q (JM) [109]
